# Coenzyme Q_10_: Clinical Applications beyond Cardiovascular Diseases

**DOI:** 10.3390/nu13051697

**Published:** 2021-05-17

**Authors:** Lara Testai, Alma Martelli, Lorenzo Flori, Arrigo F. G. Cicero, Alessandro Colletti

**Affiliations:** 1 Department of Pharmacy, University of Pisa, 56120 Pisa, Italy; alma.martelli@unipi.it (A.M.); lorenzo.flori@phd.unipi.it (L.F.); 2 Interdepartmental Research Centre ‘‘Nutraceuticals and Food for Health (NUTRAFOOD)’’, University of Pisa, 56120 Pisa, Italy; 3 Interdepartmental Research Centre of Ageing, Biology and Pathology, University of Pisa, 56120 Pisa, Italy; 4 Medical and Surgical Sciences Department, University of Bologna, 40138 Bologna, Italy; arrigo.cicero@unibo.it; 5 Italian Nutraceutical Society (SINut), 40138 Bologna, Italy; alessandro.colletti@unito.it; 6 Department of Science and Drug Technology, University of Turin, 10125 Turin, Italy

**Keywords:** coenzyme Q_10_, ubiquinone, neuronal and muscular degenerative disorders, cancer, prevention, supplementation

## Abstract

Coenzyme Q_10_ (CoQ_10_) is an essential cofactor in oxidative phosphorylation (OXPHOS), present in mitochondria and cell membranes in reduced and oxidized forms. Acting as an energy transfer molecule, it occurs in particularly high levels in the liver, heart, and kidneys. CoQ_10_ is also an anti-inflammatory and antioxidant agent able to prevent the damage induced by free radicals and the activation of inflammatory signaling pathways. In this context, several studies have shown the possible inverse correlation between the blood levels of CoQ_10_ and some disease conditions. Interestingly, beyond cardiovascular diseases, CoQ_10_ is involved also in neuronal and muscular degenerative diseases, in migraine and in cancer; therefore, the supplementation with CoQ_10_ could represent a viable option to prevent these and in some cases might be used as an adjuvant to conventional treatments. This review is aimed to summarize the clinical applications regarding the use of CoQ_10_ in migraine, neurodegenerative diseases (including Parkinson and Alzheimer diseases), cancer, or degenerative muscle disorders (such as multiple sclerosis and chronic fatigue syndrome), analyzing its effect on patients’ health and quality of life.

## 1. Introduction

**Figure 1:**
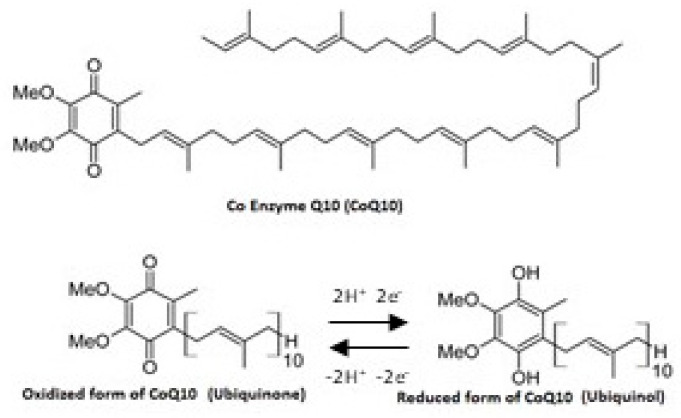
Chemical structure of CoQ_10_.

Coenzyme Q_10_ (CoQ_10_) is an organic molecule similar to a vitamin and it can even be synthesized by human cells. It consists of a benzoquinone group with a poly-isoprenoid side chain (10 units in humans) and it is generally present in cell membranes and especially in the mitochondria in both reduced (ubiquinol) and oxidized (ubiquinone) forms (Figure 1) [1]. The levels of CoQ_10_ are particularly high in the liver, kidney, and heart, organs with high metabolic activity (Table 1) [2]. Indeed, CoQ_10_ plays a key role in supplying energy to all cells and, especially, taking part in redox reactions within the electron transport chain at the mitochondrial level. In fact, this molecule is an excellent electron carrier able to support continuous oxidation-reduction cycles. Specifically, CoQ_10_ facilitates the production of adenosine triphosphate (ATP), carrying the electrons from complexes I and II to complex III of the mitochondrial respiratory chain [3,4]. In addition, CoQ_10_ is the major lipid-soluble antioxidant and it protects cell membranes and lipoproteins from oxidative damage [3]. The antioxidant activity of CoQ_10_ is linked to its reduced form, the ubiquinol, able to reduce oxidative stress, lipid peroxidation, and regenerate also vitamins C and E back to their active, fully reduced forms [5]. Last but not least, some in vitro studies have shown the ability of CoQ_10_ to reduce the inflammatory markers, suggesting that this molecule might have anti-inflammatory action through the regulation of gene expression [6,7]. However, some factors, such as aging, drugs (e.g., statins), genetic factors, neurodegenerative diseases, and degenerative muscle disorders, are well-known to be associated with reduced plasma concentrations of CoQ_10_ [5], resulting in exacerbation of oxidative stress and inflammatory processes. This takes place through the upregulation of nuclear factor kappa-light-chain-enhancer of activated B (NF-κB) gene expression and chronic activation of immune inflammatory responses [8].

The largest percentage CoQ_10_ is synthetized in the cell (4-hydroxybenzoate is the precursor of a quinone ring, while an isoprenoid tail is derived from the mevalonate pathway), although the pathways involved are not yet completely known. It is a complex multistage process (governed by at least 13 genes), requiring a number of vitamins, amino acids, trace element precursors, and cofactors, and a deficiency of any of which can adversely affect the normal CoQ_10_ production [9,10].

Furthermore, CoQ_10_ can be derived from the diet (about 5 mg/day for a Mediterranean diet). In particular it is present in fatty fishes, soja, nuts, and spinach [11], however, its intake may not be sufficient to counteract physiological or pathological deficiencies [12]. For this reason, nutritional supplementation with this nutraceutical could help to maintain adequate levels within the body.

For its important role in the organ function, a deficit of plasma levels of CoQ_10_ is associated with many degenerative states and several diseases such as cardiovascular and cerebrovascular diseases (e.g., heart failure, myocardial infarction, migraine, chronic kidney disease, and hypertension), Alzheimer (AD) and Parkinson diseases (PD), muscular dystrophy, and others [13]. However, data concerning CoQ_10_ supplementation beyond the cardiovascular field is still limited and often conflicting. Therefore, this review aims to discuss the potential applications of CoQ_10_ in various conditions with an analysis of its impact on patients’ health and quality of life.

## 2. Bioavailability of CoQ_10_

**Figure 2:**
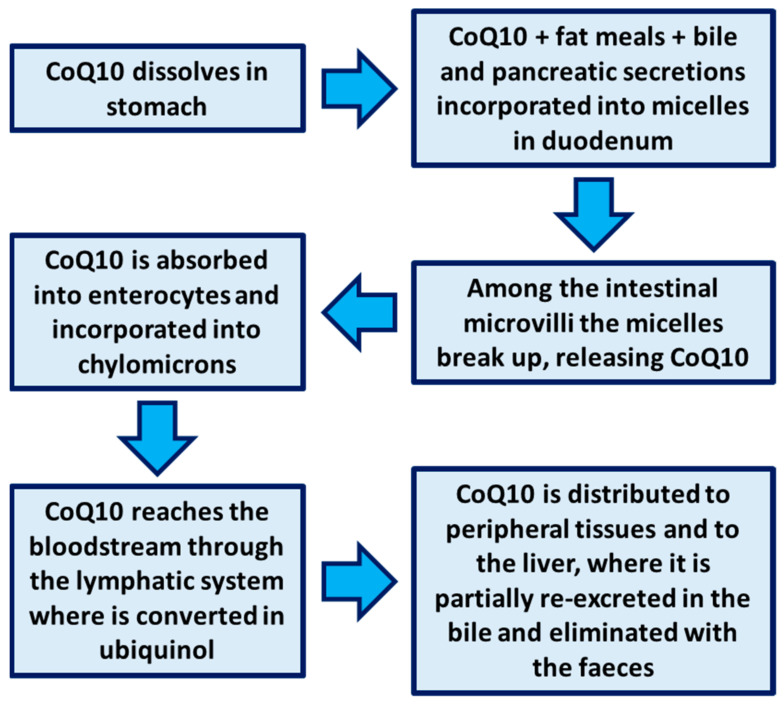
Coenzyme Q_10_ physiology.

CoQ_10_ has a very low and variable oral bioavailability, depending on many factors such as the dosage of administration, the type of formulation, the release method, and the mode of administration (e.g., before, during or after meals) [16]. Indeed, if administered in fed state, CoQ_10_ arrives in the intestinal lumen with exogenous cholesterol, then is taken up from the mixed micelles, together with meal fats, bile, and pancreatic secretions, which facilitate its solubilization and the entrance into enterocytes via the “simple passive facilitated diffusion” (“passive” because energy consumption is not required and “facilitated” because a lipid carrier, usually a monoglyceride fat, makes possible the intestinal transport). CoQ_10_, incorporated in the chylomicrons in the enterocytes, subsequently reaches the blood through the lymphatic system. From the bloodstream, CoQ_10_ is distributed to peripheral tissues and to the liver, where it is partially re-excreted in the bile and eliminated with the feces (Figure 2) [17]. The main limiting factor of low bioavailability of CoQ_10_ (molecular weight = 863) is its poor solubility in gastrointestinal fluids. For this reason, various formulations have been proposed in the market in the form of tablets, capsules, chewable tablets, and gels containing oily suspensions to improve CoQ_10_ bioaccessibility and bioavailability. The reduction of particle size (e.g., the use of nanoparticles) and the setup of biopharmaceutical strategies like the use of the β-cyclodextrin complex, liposomes, emulsions, nanostructured lipid carriers, and micelles have already been demonstrated to improve the CoQ_10_ bioavailability with satisfactory results [18,19].

## 3. Methods

The literature search has been performed using the most relevant databases recognized for medical scientific literature, including PubMed, MEDLINE (National Library of Medicine, Bethesda, Maryland, MD, USA; January 1970 to February 2021), and the Cochrane Register of Controlled Trials (The Cochrane Collaboration, Oxford, UK) with access to Scopus, EMBASE and ClinicalTrials.gov. The terms used for the electronic search strategy were “coenzyme Q_10_,” “ubiquinol,” “ubiquinone,” “dietary supplement,” “human,” and “clinical trial.” The eligible papers must be published in English. The selected references were further screened for applications beyond the cardiovascular field. Randomized clinical trials were preferred and included wherever possible, although in some minor conditions open-label studies were considered due to the lack of controlled studies. The work is characterized by a first general introduction regarding the pharmacodynamic profile and the properties of CoQ_10_ followed by a brief description of the mechanism of action for each potential therapeutic area, the effects observed in clinical studies, and the respective tolerability notes. The Declaration of Interest forms related to real or potential sources of conflicts of interest were compiled by the drafting and review groups.

## 4. Results

### 4.1. CoQ10 and Migraine

Migraine shows a prevalence of 11.6% in the world. Acute attacks of unilateral throbbing headache (lasting for 4–72 h), photophobia, phonophobia, osmophobia, nausea, vomiting, cranial allodynia, and movement sensitivity are more frequently referred and, inevitably, associated to worsening of life quality. Of note, a deficiency of CoQ_10_ is associated with the pathogenesis of migraine, especially in the pediatric and adolescent populations. Indeed the American Academy of Neurology guidelines recommended a supplementation with CoQ_10_ in migraine prevention (level of evidence C) [20].

In this regard, a randomized double-blind placebo controlled clinical trial, carried out on 45 patients enrolled in two arms: 22 treated with placebo and 23 treated with CoQ_10_ (400 mg/day), highlighted a significant prophilactic effect of the supplementation on migraine attacks after 3 months. Interestingly, less severe, shorter, and less frequent attacks were reported. Moreover, higher levels of CoQ_10_ and lower levels of tumor necrosis factor α (TNFα) and calcitonin gene-related peptide (GCPR) were measured in the serum of patients enrolled in the CoQ_10_-arm, suggesting an effect through the mitigation of inflammatory processes [21]. 

Such results have been confirmed in a systematic review and meta-analysis in which four randomized clinical trials were analyzed. CoQ_10_ supplementation significantly reduced the frequency of migraine attacks, although no significant effect on severity and duration has been reported [22]. Unlike, other studies observed a not significant reduction of frequency and severity of attacks; however, a considerable heterogeneity of the dose of CoQ_10_ and the clinical condition of patients could represent an important limitation [23].

Encouraging results emerge when CoQ_10_ (400 mg/day) is associated with other nutraceuticals typically used for the prophylaxis of migraine, such as curcumin, magnesium, and *Tanacetum parthenium* L. or riboflavin [24,25]. 

### 4.2. CoQ_10_ and Fatigue

Fatigue is a generic term which refers to multiple aspects of human physiology. Acute fatigue has been described as “reversible motor weakness and whole-body tiredness that were predominantly brought on by muscular exertion and was relieved by rest.” Chronic fatigue is a consequence of acute fatigue accumulation, and in some cases, it may be irreversible [26]. Another classification of fatigue concerns “physical” and “mental” fatigue [27]. Moreover, it can also be felt as a pervasive sensation in several diseases such as fibromyalgia, cancer, chronic fatigue syndrome (CFS), anemia, human immunodeficiency virus (HIV), and multiple sclerosis (MS) [28]. Patients reported a lack of energy to perform their daily work and fatigue during rest [29]. Considering this, CoQ_10_ could be a valid nutraceutical supplement for its multiple functions, starting from the key role in energy metabolism, but also for its antioxidant properties [30]. In general, as highlighted by the meta-analysis of RCTs by Mehrabani et al., the therapeutic effects of CoQ_10_ were better in patients with statin-related fatigue and fibromyalgia when compared with the other disease-related fatigue [28]. However, more clinical trials with sufficient follow-up periods and with adequate sample sizes are required. The potential use of CoQ_10_ in the treatment of different aspects of fatigue is described below.

#### 4.2.1. CoQ_10_ in Patients with Chronic Fatigue Syndrome 

CFS is a severe, complex, and highly weakening chronic condition. Today its causes are unknown, as well as diagnostic tests and effective treatments, currently, are focused on the control of disease symptoms. It is characterized by protracted, debilitating, and relapsing fatigue, often accompanied by other symptoms, with significant disability for at least 6 months and often for years [31]. Recent studies revealed that oxidative stress and mitochondrial dysfunction can be associated with its pathogenesis and a reduced rate of ATP synthesis has been reported [32,33,34]. Moreover, clinical reports advise an important role for mitochondrial dysfunction-dependent events and oxidative damage at the cellular level [35]. As CoQ_10_ and nicotinamide adenine dinucleotide (NADH) increase cellular ATP production through mitochondrial OXPHOS, their supplementation in fatigue, as well as in other symptoms in CFS, is considered a new alternative and complementary therapy [36,37]. 

Castro-Marrero’s group designed a clinical trial on 73 Spanish subjects (NCT02063126) in which a significant fatigue improvement, measured as decrease in fatigue impact scale (FIS) total score, was observed in the group treated with CoQ_10_ (200 mg/day) + NADH (20 mg/day) versus placebo. Moreover, a recovery of the biochemical parameters was also reported. In particular, CoQ_10_, NAD^+^/NADH ratio, citrate synthase activity, and ATP levels were significantly improved, and the cellular indicators of oxidative stress lipoperoxides, were markedly reduced in mononuclear blood cells of the treated group. Then, the combination of CoQ_10_ + NADH caused a significant reduction in fatigue, oxidative damage, enhancement of mitochondrial function and improvement of energy [34], leading to the possibility that oral supplementation with CoQ_10_ + NADH could be beneficial to treat fatigue and act on biochemical parameters in CFS. 

More recently, the same authors carried out a further RCT on 80 CFS patients, showing that CoQ_10_ + NADH supplementation was well tolerated and safe and reduced maximum heart rate significantly at week 8 versus baseline during a cycle ergometer test, supposing that at the basis of this there was an improvement of the endothelial function. Perception of fatigue also revealed a decrease during all follow-up visits in active group versus placebo, but no change in pain and sleep was observed [38].

Finally, Fukuda et al. did not support the efficacy of CoQ_10_ supplementation (150 mg/day) for 8 weeks in patients with CFS; indeed, no improvement of clinical symptoms was observed. However, the authors reported that a 12-weeks treatment with CoQ_10_ (150 mg/day) was beneficial for improving sleep quality and autonomic nervous dysfunction as well as better performance on the arithmetic task when compared with the placebo group. Interestingly, the decreases in fatigue levels and depression level were strictly correlated with the increases in ubiquinol concentrations [39]. 

#### 4.2.2. CoQ_10_ in Patients with Fibromyalgia 

The etiology of fibromyalgia is described by different pathophysiological processes such as bioenergetics alteration, mitochondrial dysfunction, oxidative stress, and inflammatory cascades. 

AMP-activated protein kinase (AMPK) plays a key regulatory function in all cascades [40,41]. AMPK is an enzyme that contributes to keeping correct cellular energy homeostasis and is down regulated in fibromyalgia patients [42,43]. AMPK activation was induced by CoQ_10_, resulting in an improvement in clinical symptoms in fibromyalgia patients [44,45]. 

Four RCTs and one quasi-experimental study have examined the effect of CoQ_10_ supplementation in people with fibromyalgia. A quasi-experimental study conducted by Cordero et al. 2013 on 35 females treated with CoQ_10_ at a dose of 300 mg/day or placebo for 3 months showed a significant reduction in fibromyalgia fatigue (assessed by Fibromyalgia Impact Questionnaire (FIQ) and Visual Analogue Scale (VAS) [46]. Similar results were obtained by the same research group in a RCT of 20 female patients treated for 40 days with 300 mg/day of CoQ_10_ (Cordero et al., 2013). Even Miyamae et al. showed an improvement of juvenile fibromyalgia in patients after 3 months of treatment with 100 mg/day ubiquinol (Chalder’s Fatigue Scale significantly reduced if compared to the control) [47]. Finally, significant improvements in FIQ and VAS were obtained in two other studies including female patients with fibromyalgia (dosages of CoQ_10_ 300–400 mg/day) [40,41].

#### 4.2.3. CoQ_10_ in Patients with Statin-Associated Myopathy 

The mechanisms involved in statin-associated myopathy are still unclear, but presumably involve an increase in intracellular lipid production and lipid myopathy and myocellular phytosterols, decrease in sarcolemmal cholesterol, reduction in mitochondrial CoQ_10_ and in small guanosine triphosphate-binding proteins [48]. Statin drugs act by inhibiting hydroxyl-methylglutaryl coenzyme A (HMG-CoA) reductase. This enzyme controls not only the synthesis of cholesterol but also the synthesis of farnesyl pyrophosphate, that is necessary for CoQ_10_ production and could explain the link between statins and CoQ_10_ deficit [49].

Only two clinical trials have examined the impact of CoQ_10_ supplementation against fatigue in statin-associated myopathy patients. Fedacko et al. highlighted a marked improvement of VAS in patients with statin-associated myopathy treated with 200 mg/day CoQ_10_ for 3 months compared to the control [50]. Another study conducted on 50 patients who followed a discontinuous therapy with statin and supplemented with 240 mg/day CoQ_10_ for 22 months, showed that the incidence of fatigue decreased from 84%, observed at the beginning of the treatment, to 16% at the end of the study [45].

#### 4.2.4. CoQ_10_ and Fatigue in Healthy Volunteers 

The action CoQ10 supplementation had on fatigue was examined in healthy subjects in 4 clinical trials. In the RCT by Mizuno et al., 17 healthy volunteers were randomized to receive CoQ_10_ at a dosage of 100 or 300 mg/day or placebo during 8 days of activity-induced fatigue. The measurement of subjective fatigue was performed through VAS. The analysis carried out with this tool revealed a significant fatigue reduction in the group treated with the highest dosage of CoQ_10_ (300 mg/day) when compared with the control group [27]. However, in 3 other studies regarding on 16 soccer players [51], 51 obese subjects [52] and 15 sedentary man [53], CoQ_10_ administration (100–300 mg/day for period of 1 to 3 months) failed to show any beneficial effects on fatigue reduction during exercise or perceived fatigue. One potential motive for the inefficiency of CoQ_10_ supplementation against fatigue in healthy subjects might be due to the baseline and change percentage of CoQ_10_ plasma levels. In particular, to increase the plasma ubiquinol level and exert a beneficial effect on fatigue, at least 300 mg/day of CoQ_10_, seems to be necessary [27,54].

#### 4.2.5. CoQ_10_ and Elite Athletes

Athletes subjected to repeated intense physical training are more susceptible to a condition of chronic fatigue, including overtraining syndrome and infectious diseases, thus compromising their performance. In fact, it is well-known how intense exercise stimulates inflammatory responses, moreover reactive oxygen species (ROS) increases, leading to oxidative stress [55]. Inflammation and oxidative stress can contribute to overtraining syndrome and chronic fatigue in athletes. Moreover, it is well-known that intensive physical exercise may also cause muscular injury [56].

Therefore, the use of antioxidant supplementations is considered as a strategy to strengthen the defenses during training and competition.

Several studies have found that, after exercise in trained athletes and untrained individuals, supplementation with CoQ_10_ improved the aerobic power, anaerobic threshold, exercise performance, and/or recovery [57]. Conversely, other studies have shown no ergogenic benefit in untrained and trained individuals on maximal or submaximal exercise capacity [58].

In a 22-days double-blind study, 120 mg/day of CoQ_10_ also increased the anaerobic work, but no differences was reported between groups in running maximal oxygen consumption (VO2max) or heart rate and rate of perceived exertion during submaximal work rates. However, the CoQ_10_ group showed significantly higher levels of creatine kinase activity, possibly for increasing free radical formation. Therefore, the authors of the paper do not recommend the use of CoQ_10_ in the athletes [59]. 

On the other hand, a blinded study demonstrated that 18 male adolescent swimming athletes supplemented for 28 days with CoQ_10_ (100 mg/day), showed significantly elevated levels of it in the blood and a positive correlation with VO2max [60]. Similar results have been obtained on endurance athletes (male road cyclists and triathletes) [61].

Greenwood et al., observed that a supplementation for 2 weeks with 200 mg/day of CoQ_10_ increased the total CoQ_10_ plasma concentration. Moreover, acute supplementation with CoQ_10_ resulted in higher CoQ_10_ concentration in skeletal muscles and lower serum oxidative stress during and following exercise; this effect was probably due to a combination of enhanced OXPHOS within the mitochondria and/or enhanced antioxidant protection [30].

Kendo exercise is also highly intense and then may cause an increase in oxidative stress and cellular damage [62,63]. Interestingly, 300 mg/day of CoQ_10_ supplementation, for 2 weeks, in 18 elite athletes enrolled in a randomized clinical trial, showed increased CoQ_10_ levels in the blood, lower creatine kinase activity and myoglobin concentration when compared with the placebo group, suggesting that it could be useful for reducing exercise-induced muscle damage [63].

Recently, a double-blind 2-weeks long clinical trial carried out on 18 male elite kendo athletes demonstrated that 300 mg/day of CoQ_10_ contributed to down-regulate the expression of Toll-like receptor 4 (TLR4), considered as an event downstream the ROS production and responsible for immune system regulation. Therefore, in athletes undergoing intensive exercise, continuous CoQ_10_ supplementation is recommended 14 weeks before starting the training in order to inhibit exercise-induced inflammation and immune deficiency [64]. 

Moreover, 14-day CoQ_10_ supplementation (5 mg/Kg/day), significantly improved antioxidant and anti-inflammatory defenses following training such as running (i.e., competitive 3000 m) [65]. 

Furthermore, a previous study on 25 skiers highlighted higher levels of CoQ_10_ in the blood of athletes treated with this supplementation after 6 weeks of training [54].

Very recently, Emami et al., reported that in 36 healthy elite swimmers, CoQ_10_ supplementation prevented changes in mediators of inflammatory cytokines following heavy swimming trainings and acute recording bout [66]. 

#### 4.2.6. CoQ_10_ in Patients with Other Fatigue-Related Diseases 

Patients awaiting cardiac transplantation with end-stage heart failure experienced fatigue in carrying out daily activities, such as dressing or teeth brushing. The treatment for 3 months with CoQ_10_ at a dose of 60 mg/day has been reported to significantly reduce fatigue symptoms [67].

Nevertheless, the RCT by Peel et al., showed that the administration of 100 mg/day CoQ_10_ for 2 months failed to alleviate fatigue in the late-onset sequelae of 101 patients with poliomyelitis [68]. Similarly, in the last RCT, CoQ_10_ supplementation did not show any significant efficacy in fatigue reduction on newly diagnosed patients (236 females) with breast cancer [69]. 

### 4.3. CoQ_10_ and Neurodegenerative Diseases

#### 4.3.1. CoQ_10_ and Parkinson Disease

The neurodegenerative diseases (ND) are related to a persistence of neuroinflammation processes and free-radicals generation which leads to cellular oxidative stress mainly due to mitochondrial dysfunction. In this panorama, the recognized role of CoQ_10_ as an antioxidant agent endowed with anti-inflammatory properties has been suggested in last years as a promising strategy to be associated with the therapy of ND [5]. Concerning PD, in in vitro and in vivo pre-clinical studies, CoQ_10_ demonstrated a neuroprotective activity on nigrostriatal dopaminergic neurons [70], while clinical evidences highlighted low levels of CoQ_10_ in mitochondria of parkinsonian patients [71,72]. On this basis, several clinical trials have been carried out on CoQ_10_ supplementation in PD. Müller et al., in a placebo-controlled double-blind trial on 28 patients treated for four weeks with 360 mg/day of CoQ_10_, observed a significant mild symptomatic benefit in CoQ_10_-treated patients compared with placebo-treated parkinsonian patients [73]. In contrast, Shults and colleagues, in a 16-months-long randomized, placebo-controlled, double-blind trial on 80 patients receiving several doses of CoQ_10_ (300, 600, and 1200 mg/day), found that a supplementation of CoQ_10_ 1200 mg once a day was able to reduce the worsening in early PD, with great improvements in patients daily routine activities such as feeding, bathing, or dressing. Moreover, the same trial recorded also an improvement in NADH-cytochrome c reductase activity and an increase in CoQ_10_ plasmatic levels [74]. High doses were reported also by Horstink et al., in an open label trial on a limited number of patients (*n* = 12) who received 1000 mg/day of CoQ_10_ for three months and then 1500 mg/day of CoQ_10_ for other three months. At the end of the trial, lower but statistically significant benefits on motor performance in patients who received 1500 mg/day vs. placebo were recorded [75]. In order to assess tolerability of high doses, in 2004, Shults et al., performed an open label study on 17 patients who received growing CoQ_10_ dosages from 1200 mg to 3000 mg/day over 8 weeks. The results were that 13 of those patients tolerated the highest dose but their plasma levels reached a plateau at 2400 mg/day, indicating the futility to administer higher doses [76]. At the same time, a multicenter, randomized, placebo-controlled, double-blind study was carried out by Storch and colleagues to establish if the CoQ_10_ effects on PD may be neuroprotective or symptomatic. For this purpose, 106 parkinsonian patients with no motor complications and stratified by treatment with levodopa, received 300 mg/day of CoQ_10_ for three months. The study had, as its primary endpoint, changes in Unified Parkinson’s Diseases Rating Scale (UPDRS), but despite that serum levels of CoQ_10_ were increased in all patients in a way similar to the administration of 1200 mg/day performed by Shults et al., in 2002, no significant changes in UPDRS were observed, suggesting that in a mid-stage of PD, the CoQ_10_ supplementation does not induce symptomatic effects [77]. On the basis of these studies, more recently, the Parkinson Study Group QE3 investigators carried out a phase III RCT on 600 patients from North America, who received placebo, 1200 mg/day or 2400 mg/day of CoQ_10_, as well as all the patients received 1200 IU/day of vitamin E (vit E). After 16 months of treatment, the primary outcome was the total UPDRS score was analyzed but no significant differences were found between patients treated with placebo or the two doses of CoQ_10_ [78]. A following interesting study focuses attention on the preclinical models which seem to suggest that the reduced form of CoQ_10_ (ubiquinol-_10_) was more effective in a mouse model of PD than the oxidized form of CoQ_10_. Moreover, higher plasma concentrations of CoQ_10_ have been recorded after ingestion of the reduced form vs. the oxidized form [79]. According to this hypothesis, a randomized, double-blind, placebo-controlled, parallel-group pilot trial was carried out on Japanese patients to assess the efficacy of 300 mg/day of ubiquinol-_10_ or placebo for 48 weeks in patients experiencing wearing off, or 96 weeks in early PD patients not treated with levodopa. Results indicated that ubiquinol-_10_ induced a significant decrease in UPDR scores, showing the ability to improve PD patients experiencing wearing off, but it was not able to induce an improvement in early PD patients [80].

#### 4.3.2. CoQ_10_ and Huntington Disease

The history of research about a possible role of CoQ_10_ in Huntington disease (HD) therapy/supplementation started with a small pilot trial in 1996 on 10 patients. During these 6 months long open-label trial, patients receiving from 600 to 1200 mg/day of CoQ_10_ were evaluated according to the HD Rating Scale (HDRS), the HD Functional Capacity Scale (HDFCS), and standardized neuropsychological measures. The study highlighted a good tolerability of CoQ_10,_ but no significant improvement of HD according to the above clinical scores was recorded. A wider study, the CARE-HD trial, was carried out by the Huntington Study Group on 174 patients with early HD. During this randomized, placebo-controlled, multicenter, double-blind trial, the patients received CoQ_10_ 300 mg twice daily or remacemide 600 mg daily for 30 months, but no significant changes in total functional capacity (TFC) were observed both for CoQ_10_ and for remacemide treated patients [81]. More recently, Mc Garry and colleagues performed a multicenter randomized, double-blind, placebo-controlled trial on 609 early HD patients from the United States, Canada, and Australia. HD patients received placebo or CoQ_10_ 2400 mg/day for 60 months. Variations in total functional capacity score from baseline to 60th (for patients who survived), combined with time to death (for patients who died), was used as the primary outcome variable in using a joint-rank analysis approach. Even in this study no significant changes in TFC were recorded, leading the authors to assert that on the bases of their results, use of CoQ_10_ is not justified in a therapy focused on the slowing down of functional decline in HD [82]. 

#### 4.3.3. CoQ_10_ and Alzheimer Disease 

Data about a possible role of CoQ_10_ in the treatment of AD is still lacking and the only clinical trial reported about this topic is a double-blind, placebo-controlled trail, carried out on 78 patients with mild/moderate AD. The patients were randomized into three groups receiving a 16-week long treatment with 400 mg of CoQ_10_ three times/day or an anti-oxidant cocktail composed of 800 IU/day of vit E + 500 mg/day of vitamin C + 900 mg/day of α-lipoic acid (E/C/ALA) or placebo. The main outcomes were represented by changes from the baseline values of cerebrospinal fluids (CSF) biomarkers related to AD and oxidative stress, of cognitive parameters (through the Mini-mental state examination, MMSE) and functional ability (through the Alzheimer’s Disease Cooperative Study Activities of Daily Living Scale). The results of the study reported that CSF F2-isoprostane levels and oxidative stress biomarkers decreased by about 19% in the E/C/ALA group but at the same time an accelerated decline in MMSE scores was recorded for the same E/C/ALA group, while no significant changes occurred in the CoQ_10_ group both for CSF biomarkers and in MMSE scores. Finally, no significant changes in CSF Aβ42, tau, and P-tau (181) levels were observed in all the three groups [83].

#### 4.3.4. CoQ_10_ and MS

A degenerative condition in which fatigue is a typical symptom, besides depression, is MS [84]. MS-related fatigue appears when heat intensifies during daytime [85]. Although the exact etiology of fatigue is still unknown, multifactorial causes such as endocrine and autoimmune disorders are involved. Furthermore, there is no connection with the degree of impairment of the nervous system, disability, or MS [86]. In particular, inflammatory processes seem to be involved in these symptoms; indeed, pro-inflammatory cytokines, such as TNF-α, are associated with anorexia, weight loss, locomotors retardation, anxiety, and decreased social exploration [87]. On the other hand, major depression has a strong relationship with inflammation, showing an increase also in oxidative stress markers such as malondialdehyde (MDA) [88]. Regarding a possible role played by CoQ_10_, a unique paper has been published in 2015 by *Sanoobar and colleagues*. They carried out a randomized, double-blind, placebo-controlled clinical trial on 48 patients with relapsing-remitting MS in order to evaluate the effect of a CoQ_10_ supplementation (500 mg/day). After 12 weeks, depression and fatigue were improved. In particular, fatigue was measured by FSS and depression was evaluated through the Beck Depression Inventory (BDI). The results of this study highlighted a significant decrease of FSS and BDI in the CoQ_10_ group compared to the placebo group, demonstrating a possible beneficial effect of CoQ_10_ supplementation on fatigue and depression associated with MS [89]. Moreover, the authors found that the levels of inflammatory markers TNF-α, interleukin 6 (IL-6), and matrix metallo-proteinase 9 (MMP-9) were significantly decreased in the CoQ_10_-treated group, while the supplementation did not affect the levels of the anti-inflammatory markers interleukin 4 (IL-4) and tissue growth factor β (TGF-β), hypothesizing that at the basis of these beneficial effects there are antioxidant and anti-inflammatory activities [89].

### 4.4. CoQ_10_ and Neuropathy

#### 4.4.1. CoQ_10_ and Diabetic Neuropathy

As concerns neuropathy, a 12-week RCT focused on diabetic neuropathy was carried out on 70 patients with type 2 diabetes who received 200 mg/day of CoQ_10_ or placebo for 12 weeks. At the end of the trial hemoglobin glycate (HbA1c), fasting blood glucose and lipid profiles did not show significant differences between the two groups. On the other hand, in the group treated with CoQ_10_, a significant increase of the mean insulin sensitivity and the total antioxidant capacity concentration was recorded. Moreover, high sensible C-reactive protein (hsCRP) levels were found significantly decreased in the CoQ_10_ group compared to placebo. Despite a clear improvement of the biochemical markers, the evaluation of neuropathic symptoms and electromyography measurements did not highlight significant differences between the two groups after the trial [90].

#### 4.4.2. CoQ_10_ and Glaucoma

Glaucoma is a progressive optic neuropathy which may benefit from a neuroprotective strategy. In this context, 43 open-angle glaucoma patients were treated for 12 months with CoQ_10_ in association with vit E eye drops formulation in addition to β-blockers or with β-blockers monotherapy. Interestingly, patients who received 100 mg CoQ_10_ + 500 mg vit E + β-blockers, compared to those only treated with β-blockers, showed a beneficial effect on inner retinal function, electroretinogram improvement and enhancement of the visual cortical responses (measured by visual-evoked potential enhancement) [91]. Moreover, in a prospective randomized clinical study on pseudo-exfoliative glaucoma, 64 patients underwent phacoemulsification and intraocular lens implantation surgery, which was carried out while administering topically 100 mg CoQ_10_ + 500 mg vit E TPGS eye drops + prostaglandin agent twice daily for one month in a pre-operative phase or only the prostaglandin agent. As results of this study, the authors recorded aqueous humor-superoxide dismutase levels significantly lower in the group treated with the addition of CoQ_10_ + VitE than in the group receiving only the prostaglandin agent, while no significant differences were observed in malondialdehyde levels between the groups [92].

### 4.5. CoQ_10_ and Cancer

#### 4.5.1. CoQ_10_ and Breast Cancer

One of the first studies on the supplementation with CoQ_10_ in breast cancer was a randomized clinical trial on 84 patients who received one dosage per day of a complex named “CoRN,” composed of 100 mg of CoQ_10_, 10 mg of riboflavin, and 50 mg of niacin in addition to 10 mg of tamoxifen twice a day. To evaluate the efficacy of the treatment, as primary outcomes, two markers of circulating breast cancer, carcinoembryonic antigen (CEA) and carbohydrate antigen 15–3 (CA 15–3), were recorded. Moreover, serum cytokine levels (IL-1β, IL-6, IL-8, TNF-α) and vascular endothelial growth factor (VEGF) were also recorded. The levels of the two markers and of cytokine were found elevated in untreated patients and significantly reduced in the patients who received tamoxifen for more than 1 year. Interestingly, CEA, CA 15–3, and serum cytokine were further reduced in patients who, in addition to tamoxifen, received CoRN for 45 days or 90 days demonstrating that CoRN supplement could help chemotherapy to control the risk of metastases [93]. In the same study a significant increase in DNA repair enzymes (poly-ADP-ribose polymerase levels), a disappearance of DNA methylation patterns (RASSF1A DNA methylation pattern), and a reduction of pro-angiogenic marker levels were observed, suggesting an improvement of the prognosis [94,95].

More recent studies focused on a particular aspect concomitant with breast cancer that is fatigue associated with this pathology. In a first randomized, double-blind, placebo-controlled study, 236 women newly diagnosed and who planned adjuvant chemotherapy received CoQ_10_ 300 mg or placebo, each associated with 300IU vit E in three daily doses, for 24 weeks. At the end of the study, the authors found that, despite the increase of CoQ_10_ levels, no significant differences were found in scores obtained through the Profile of Mood States-Fatigue questionnaire, Functional Assessment of Chronic Illness Therapy-Fatigue tool, Functional Assessment of Cancer Therapy-Breast Cancer instrument, or Center for Epidemiologic Studies-Depression scale [96]. On the other hand, a randomized clinical trial carried out in Japan on 57 breast cancer patients with cancer-related fatigue (CRF) undergoing chemotherapy, receiving the amino acid jelly Inner Power(^®^) (IP), an oral dietary supplement containing branched-chain amino acids (2500 mg), CoQ_10_ (30 mg), and l-carnitine (50 mg), or regular care for 21 days, reported significant differences between the two arms in the scores of worst level of fatigue, global fatigue scores, and current feeling of fatigue. While non-significant value were recorded with regard to Hospital Anxiety and, Depression Scale and the European Organization for Research and Treatment of Cancer Quality of Life Questionnaire Core 30, concluding that IP may control moderate-severe cancer-related fatigue in breast cancer [97].

#### 4.5.2. CoQ_10_ and Hepatocellular Carcinoma (HCC)

HCC is characterized by higher levels of oxidative stress and inflammation and this condition persists also during its progression after surgery. On this basis *Liu and colleagues* performed a 12 weeks single-blinded, randomized, parallel, placebo-controlled study on 41 patients diagnosed with primary HCC after surgery consisting of tumor resection, biopsy, and classification of their tumor as primary HCC according to the International Classification of Diseases 9, code 155.0. After this diagnosis, the enrolled patients were treated randomly with placebo or CoQ_10_ 300 mg/day. At the end of the study it was found that oxidative stress and inflammatory markers (hs-CRP and IL-6) were significantly decreased with a contemporary increase in the activity of antioxidant enzymes, including superoxide dismutase (SOD), catalase (CAT), and glutathione peroxidase (GPx) in HCC patients after surgery [96]. 

#### 4.5.3. CoQ_10_ and Prostatic Carcinoma

A randomized clinical trial on 80 patients with hormonally untreated prostatic carcinoma was carried out to assess the effect of a daily supplementation containing CoQ_10_ (2 capsules 2 × 100 mg/day), vitamin C 750 mg, selenium 200 μg, and vit E 350 mg. During the 21 weeks of the study the treated arm was compared with placebo and serum levels of prostatic specific antigen (PSA) and were assessed at baseline and after 6, 13, 19, 20, and 21 weeks. Moreover, also the mean changes in serum level of testosterone, dihydrotestosterone (DHT), luteinizing hormone (LH), and sex hormone binding globulin (SHBG) were recorded. At the end of the study, in the patients who received the supplementation, plasma levels of CoQ_10_, vit E, and selenium were significantly increased. However, no significant differences were found in serum levels of PSA, testosterone, DHT, LH, and SHBG between the two arms, concluding that in this condition the supplement did not affect the main parameters used to control the progression of prostatic cancer [98].

#### 4.5.4. CoQ_10_ and Melanoma

Currently, early surgery remains the best therapy for melanoma and there is no standard adjuvant therapy for this type of cancer. However, low serum concentration of CoQ_10_ was found in melanoma patients [99]. On this basis, *Rusciani and colleagues* carried out a 3-year trial during which 81 patients with stage I and II melanoma and surgically removed lesions received low doses of recombinant interferon α-2b (9 000 000 000 IU weekly) only, or a combination of interferon α-2b (9 000 000 000 IU weekly) and CoQ_10_ (400 mg/day). The main outcome was the incidence of recurrence at 5 years and, despite that a survival study could not be undertaken due to the small number of patients and the short duration, the authors observed a significant decrease in rates of recurrence and negligible adverse effects in the arms which received the supplementation of CoQ_10_ in addition to low doses of interferon α-2 [100].

### 4.6. CoQ_10_ and Fertility

Infertility is caused by several factors in about 30–50% of couples, and in this regard a growing body of evidence points toward oxidative stress as a deleterious aspect on spermatozoa, because of peroxidation and DNA damage [101]. It is noteworthy that CoQ_10_ is found to be involved in energy production in spermatozoa and in the defense from free radical production. 

Indeed, Eroglu et al., showed that CoQ_10_ supplementation had a positive effect on sperm morphology but not on its concentration and life-span [102]. Lower levels of CoQ_10_ have been correlated with abnormal spermatozoa. In contrast, previous results failed to find a correlation between seminal levels of CoQ_10_ and sperm motility. Balercia et al., in a randomized clinical trial, reported that 200 mg/day of supplementation for 6 months in infertile men with idiopathic asthenozoospermia showed an increased sperm motility by the end of therapy [103].

Likewise, Sefarinejad and colleagues reported that sperm morphology, motility, and density were improved in men with oligoasthenoteratozoospermia following 26 weeks of treatment with CoQ_10_ (200 mg/day) [104]. On the contrary, another randomized study demonstrated a non-significant effect on sperm morphology, motility, and concentration [105].

Previously, considering that varicocele can be a main cause of stress-oxidative-mediated infertility, also in men with normozoospermia [106], the exogenous administration of CoQ_10_ (100 mg/day) for 3 months improved the sperm density and motility together with antioxidant function [107]. In summary, although several studies have been performed on men in order to evaluate the role of CoQ_10_ supplementation on testosterone, at present the evidence is unclear and inconclusive. 

Besides male infertility, a condition that predisposes infertility in women is represented by polycystic ovarian syndrome (PCOS), that is affecting 6–20% of reproductive-aged women [108].

In this condition women generally present hyperandrogenism (in particular acne, hirsutism and alopecia), dysmenorrhea until amenorrhea, polycystic ovarian morphology, but also insulin resistance, obesity, and lipid disorders. Indeed, they are more susceptible to metabolic diseases and inflammation. On the basis of Rotterdam criteria the presence of two or three of these characteristics are necessary for a diagnosis of PCOS [109,110,111]. In this context CoQ_10_, by virtue of its antioxidant properties, is viewed as a protective agent able to improve the typical metabolic and endocrine disequilibrium.

Notable, 12 weeks of treatment with CoQ_10_ supplementation (100 mg/day) downregulated the expression of low-density lipoprotein (LDL) receptors, stimulated the AMPK, and reduced the ROS-induced endothelial damage, confirming the positive role on metabolic alterations [44].

Samimi et al., in a RCT on 60 women, confirmed the beneficial effects on cholesterol levels but also on glucose metabolism, with a supplementation with CoQ_10_ at a dose of 100 mg/day for 12 weeks [112]. Then, another RCT was developed by the same research group on 40 women suffering from PCOS, in which they received CoQ_10_ (100 mg/day) for 12 weeks. Together with the beneficial effects on glucose and lipid levels, the authors reported also a down-regulated gene expression of oxidized LDL receptor 1 and an up-regulation of peroxisome proliferator-activated receptor-γ (PPAR-γ) gene expression in PBMCs. Furthermore, CoQ_10_ supplementation in subjects with PCOS down-regulated the gene expression of IL-1, IL-8, and TNF-α in PBMCs if compared to the placebo group [113]. Izadi et al., obtained comparable results in a RCT of 85 PCOS women treated with CoQ_10_ and/or vit E or placebo. Specially, treatment with CoQ_10_ alone reduced LH and testosterone levels and improved the insulin resistance. Interestingly, the co-administration of CoQ_10_ and α-tocopherol showed a more pronounced activity and promoted the SHBG release, justifying the enhancement of insulin tolerance, which is a condition associated with a reduced hepatic synthesis of SHBG. Then, CoQ_10_, through an improvement of mitochondrial function and a restoration of energy production at mitochondrial level, can stimulate the biosynthesis of steroid hormone and normal reproductive function, like oocyte maturation, fertilization, and embryonic development [114].

### 4.7. CoQ_10_ and Dupuytren’s Disease (DD)

DD is defined as a benign progressive fibroproliferative condition of the palmar and digital fascia of the hands, resulting in irreversible flexion deformities of these. Anti-inflammatory drugs are among the most used remedies, as increased cytokine levels, especially TNF-α, have been found in patients with DD [115].

Currently, there are not any indications for supplementation with CoQ_10_ in this condition. Very recently a case report, in which a patient has been supplemented with CoQ_10_ (300 mg/day for 3 years), has been published. Indeed, the typical contracture at the hands were resolved, suggesting that CoQ_10_, by virtue of its anti-inflammatory profile, could be a valid nutritional remedy [116]. 

### 4.8. CoQ_10_ and Schizophrenia

A growing body of evidence suggests that mitochondrial dysfunction and reduced energy are at the basis of the negative and cognitive symptoms in schizophrenia, such as fatigue, isolation, and cognitive impairments. Indeed, a depletion of ATP and an increase of oxidative stress and lactate levels have been found in schizophrenic patients [117,118,119]. In addition, low levels of CoQ_10_ have been observed in the erythrocytes of patients with schizophrenia [120], therefore, a supplementation with CoQ_10_ may have a therapeutic value, but to our knowledge no clinical studies have been carried out [121].

## 5. Discussion

CoQ_10_ is an ergogenic supplement present in foods, with an excellent safety profile even with chronic exposure to 900 mg/day [122] and in frail patients, like elderly and chronic kidney disease patients, without any known pharmacological interactions [2]. Conversely, ts deficiency is associated with several pathological conditions, including, first of all, cardiovascular diseases, and its supplementation tends to improve the quality of life and in some cases, hard outcomes as well [17]. In this regard, the Q-SYMBIO study, a multicenter randomized placebo-controlled trial, showed that a daily intake of 300 mg of CoQ_10_ (*n* = 202) or placebo (*n* = 218) in patients affected by moderate or severe heart failure (HF) and treated with the conventional gold standard therapy (*n* = 420), have benefited from a significant reduction in major adverse cardiac events rate, cardiovascular mortality, all-cause mortality, and incidence of hospital stays for HF after 2 years in comparison with the placebo [123]. Despite abundant and encouraging results in the cardiovascular field (although the need for further information exists), data regarding other pathological disorders, such as neurodegenerative diseases, muscular disorders, and cancer (Table 2, Table 3, Table 4 and Table 5), are still limited and sometimes contrasting, making it difficult to arrive at definitive conclusions on the efficacy of CoQ_10_ in several diseases. However, this review aimed at examining the literature on clinical studies in which CoQ_10_ has been considered to counter non-cardiovascular pathological conditions, and aimed to highlight further possible and interesting therapeutic uses.

Indeed, CoQ_10_ can exert many mild positive effects on different tissues and metabolism through a reduction of systemic oxidative stress and inflammation, as observed in several RCTs. In this regard, a growing body of evidence suggests that CoQ_10_ exhibits significant effects on the prophylaxis of migraine, in particular when it is associated to other nutraceuticals, including curcumin, magnesium, and *Tanacetum parthenium* L. or riboflavin. Indeed the guidelines recommend it at a dose of 300 mg/day. 

CoQ_10_ has been also observed to improve the symptoms of CFS and other diseases characterized by fatigue (i.e., fibromyalgia), and also in healthy athletes submitted to intensive exercise. CoQ_10_ results generally effective in this context, although sometimes rather than parameters related to the perception of fatigue, markers of inflammation or oxidative stress are measured. Of note, in RCTs carried out on patients with CFS, CoQ_10_ has been often associated with NADH supplementation.

On the basis of the recognized role of CoQ_10_ as an antioxidant and anti-inflammatory agent, several clinical trials have been carried out on patients with ND. In MS, CoQ_10_ controls the symptoms associated to the neurological disease, such as fatigue and depression, but seems to be limited to relapsing-remitting of the MS. 

The evaluation of a potential approach with CoQ_10_ supplementation in oncologic patients highlighted a general improvement in inflammatory, oxidative, and specific biochemical markers, but unfortunately these studies are highly heterogenous, both concerning the dosages and the cocktail of drugs or supplements which oncologic patients received together with CoQ_10_. This lack of homogeneity makes it challenging to identify the real impact of CoQ_10_ supplementation on the several types of cancer and further studies should be performed to clarify its full role. 

A different evaluation could be done as concerns the use of CoQ_10_ supplementation in HD. Indeed in the last 25 years, different well-designed clinical trials have been carried out with the administration of high doses (from 300 to 2400 mg/day) of CoQ_10_ alone, in long chronic treatments (from 6 to 60 months) and trying to select patients in early stages of HD, but despite the appreciable quality of these studies, in all of them the results confirmed that no significant improvements have been recorded in HD after the administration of CoQ_10_ supplementation. This attests to the lack of encouraging findings which could suggest a CoQ_10_ application in HD therapy. Moreover, several clinical studies considered a supplementation with CoQ_10_ to improve fertility in men and women. In men, evidence is unclear and inconclusive, while in women (in particular suffering from PCOS) the supplementation with CoQ_10_ demonstrated to improve the metabolic disorder, typically associated to the disease. 

Finally data on schizophrenia, glaucoma, and DD disease are still preliminary and often inconsistent, therefore further clinical investigations are required. The reasons of the inconsistency of the data could be due to a multiplicity of factors: RCTs are frequently undersized and the period is too short to evaluate the effect on hard outcomes. Moreover, the applied methodology is generally of low-quality with an insufficient standardization of patient characteristics at the baseline. Often there is no quantification of CoQ_10_ intake with diet (even if this is usually very low) and the tested dosage is not titrated based on the blood CoQ_10_ level. Furthermore, the use of real endpoints instead of biochemical surrogated outcomes might particularly be useful in order to understand the actual value of a supplementation with CoQ_10_.

## 6. Conclusions

If, on the one hand, clinical evidence support supplementation with bioavailable-CoQ_10_ (≥200 mg/day) in the cardiovascular field, and in particular in patients affected by HF and coronary heart disease to preserve heart health. On the other hand data regarding its supplementation in people with ND, cancer, or other conditions, including glaucoma, fertility, migraine, and fatigue, are often encouraging, but need to be strengthened by long-term high-quality RCTs. In conclusion, this review paves the way for speculating on the possible supplementation with CoQ_10_ in non-cardiovascular fields, in which an intervention with such a type of nutraceuticals could furnish positive benefits.

## Figures and Tables

**Table 1:** Ubiquinone and ubiquinol distribution in human tissues.

Organ	Ubiquinone Concentration (µg/g)	Ubiquinol Concentration (µg/g)	References
Heart	132.0	61.0	[14,15]
Kidneys	77.0	75.0	
Liver	63.6	95.0	
Muscle	39.7	65.0	
Brain	13.4	23.0	
Pancreas	32.7		
Spleen	24.6		
Lung	7.9	25.0	
Thyroid	24.7		
Testis	10.5		
Intestine	11.5	95.0	
Colon	10.7		
Ventricle	11.8		
Plasma (µmol/mL)	1.1	96.0	

**Table 2:** Coenzyme Q_10_ and migraine.

	Study Design	Daily Doses	Effects on Symptoms	Effects on Lab. or Instrumental Parameters	Effects on Hard Outcomes
Migraine	RCTs	100–400 mg/day	↓ duration and severity of attacks	↓ TNFα and GCPR levels	Not investigated

↓ = it is indicative of a reduction of a marker or a symptom.

**Table 3:** Coenzyme Q_10_ and muscle-related diseases.

	Study Design	Daily Doses	Effects on Symptoms	Effects on Lab or Instrumental Parameters	Effects on Hard Outcomes
Fatigue	RCTs	200 mg/day, in association with NADH (20 mg/day)	↓ FIS total score (CFS)	↑ NAD^+^/NADH ratio and CoQ_10_, ATP, citrate synthase levels	Not investigated
	RCTs	300–400 mg	↓ FIS total score	-	Not investigated
Fibromyalgia	RCTs	100–400 mg	↓ fatigue (FIQ, VAS)	-	Not investigated
Statin-associated myopathy	Meta-analysis of RCTs	≥200 mg	↓ fatigue (VAS)	-	Not investigated
Other fatigue-related diseases	RCTs	60–500 mg	↓ fatigue (FSS) only in multiple sclerosis and in patients awaiting cardiac transplantation with end-stage heart failure	-	Not investigated

FIQ = Fibromyalgia Impact Questionnaire, FSS = Fatigue Severity Scale, RCTs = randomized clinical trials, VAS = Visual Analog Scale. ↓= it is indicative of a reduction of a marker or a symptom. ↑ = it is indicative of an increase of a marker or a symptom.

**Table 4:** Coenzyme Q_10_ and degenerative diseases.

	Study Design	Daily Doses	Effects on Symptoms	Effects on Lab or Instrumental Parameters	Effects on Hard Outcomes
PD	RCTs	300–2400 mg	↑significant mild symptomatic benefit, ↑ great improvements of patients everyday activities such as feeding, bathing, or dressing, ↑ effects on motor performance, = no significant changes in UPDRS	↑ improvement in NADH-cytochrome c reductase activity, ↑ increase in CoQ_10_ plasmatic levels	Not investigated
HD	RCTs	600–2400 mg	= no significant changes in: HDRS, in HDFCS, standardized neuropsychological measures and TFC scores	Not recorded	Not investigated
AD	RCTs	400 mg	= MMSE scores and functional ability	= not significant differences in: CSF F-2-isoprostane levels, oxidative biomarkers, CSF Aβ42, tau, and P-tau (181) levels	Not investigated
MS	RCTs	500 mg	↑reduction of fatigue and depression	↓ inflammatory markers TNF-α, IL-6 and MMP-9, = IL-4 and TGF-β levels	Not investigated
Glaucoma	RCTs	100 mg	Not evaluated	↑ inner retinal function, electroretinogram and visual cortical responses, ↓superoxide dismutase, = malondialdehyde levels	Not investigated
Neuropathy	RCTs	200 mg	No significant improvement of neuropathic symptoms	= no significant differences on HbA1c, fasting blood glucose or lipid profile, ↑mean insulin sensitivity, ↑ total antioxidant capacity concentration, ↓C-protein level, = electromyography measurements	Not investigated

CSF = cerebrospinal fluids, HDRS = Huntington’s Disease Rating Scale, HDFCS = Huntington’s Disease Functional Capacity Scale, MMSE = mini-mental state examination, NADH = nicotinamide adenine dinucleotide, RCTs = randomized clinical trials, TFC = total functional capacity, UPDRS = Unified Parkinson’s Diseases Rating Scale. ↓ = it is indicative of a reduction of a marker or a symptom. ↑ = it is indicative of an increase of a marker or a symptom.

**Table 5:** Coenzyme Q_10_ and cancer.

	Study Design	Daily Doses	Effects on symptoms	Effects on Lab or Instrumental Parameters	Effects on Hard Outcomes
Breast cancer	RCTs	300–2400 mg	↓ moderate-severe cancer-related fatigue (30 mg)	↓ CEA, CA 15-3, IL-1β, IL-6, IL-8, TNF-α, vascular endothelial growth factor, pro-angiogenic marker levels, ↑ DNA repair enzymes (poly-ADP-ribose polymerase levels), a disappearance of DNA methylation patterns (RASSF1A DNA methylation pattern)	Not investigated
HCC	RCTs	300 mg	Not investigated	↓ hs-CRP, IL-6 ↑ SOD, CAT, GPx	Not investigated
Prostatic carcinoma	RCTs	300 mg	Not investigated	↑ CoQ_10_, vit E, selenium = PSA, testosterone, DHT, LH, SBHG	Not investigated
Melanoma	RCTs	400 mg	Not investigated	Not investigated	↓ rates of recurrence at 5 years

CAT = catalase, CEA = carcinoembryonic antigen, CA 15-3 = carbohydrate antigen 15-3, DHT = dihydrotestosterone, GPx = glutathione peroxidase, hs-CRP = high sensitivity C-reactive protein, IL-1β = interleukin-1beta, IL-6 = interleukin-6, IL-8 = interleukin-8, SOD = superoxide dismutase, PSA = prostatic specific antigen, RCTs = randomized clinical trials, TNF-α = tumor necrosis factor-alpha. ↓ = it is indicative of a reduction of a marker or a symptom. ↑ = it is indicative of an increase of a marker or a symptom.
